# Impact evaluation and economic benefit analysis of a domestic violence and abuse UK police intervention

**DOI:** 10.3389/fpsyg.2023.1063701

**Published:** 2023-02-16

**Authors:** Yiannis Karavias, Siddhartha Bandyopadhyay, Christine Christie, Caroline Bradbury-Jones, Julie Taylor, Eddie Kane, Heather D. Flowe

**Affiliations:** ^1^Department of Economics, University of Birmingham, Birmingham, United Kingdom; ^2^Chanon Consulting Ltd., Epsom, United Kingdom; ^3^School of Nursing and Midwifery, University of Birmingham, Birmingham, United Kingdom; ^4^Birmingham Women’s and Children’s NHS Foundation Trust, Birmingham, United Kingdom; ^5^Centre for Health and Justice, Institute of Mental Health, University of Nottingham, Nottingham, United Kingdom; ^6^School of Psychology, University of Birmingham, Birmingham, United Kingdom

**Keywords:** intimate partner violence, evidence-based policing, Crime Harm Index, economic evaluation, batterer intervention program, domestic violence and abuse, machine learning

## Abstract

This study evaluated the impact and economic benefit of Cautioning and Relationship Abuse (CARA), an intervention which aims to reduce re-offending of first-time low-level domestic violence and abuse perpetrators. The analysis was based on two samples drawn from separate UK police force areas. CARA’s impact was assessed using a matched sample of similar offenders from a time when CARA was not available. The matching was based on a host of offender and victim characteristics and machine learning methods were employed. The results show that the CARA intervention has a significant impact on the amount of recidivism but no significant reduction in the severity of the crimes. The benefit-cost ratio in both police force areas is greater than one and estimated to be 2.75 and 11.1, respectively, across the two police force areas. Thus, for each pound (£) invested in CARA, there is an economic benefit of 2.75–11.1 pounds, annually.

## 1. Introduction

Domestic violence and abuse (DVA) is a serious and widespread problem in the UK. The Crime Survey for England and Wales (CSEW) estimates that around one in 20 people aged 16 and over experienced some form of DVA in the year 2021/2022 (Office for National Statistics, 2022a). The police are usually the first agency of contact; in the same year (2021/2022), UK police received, on average, over 70 DVA-related calls an hour, which amounts to 8% of all recorded offences (Office for National Statistics, 2022a).

In the US the situation has been similar in that DVA is frequent and to combat that, programmes that attempt to change perpetrator behavior have been introduced. Since the late 1980s, court-mandated batterer intervention programs, also referred to as “batterer interventions (BIPs),” have been widely used and studied (Feder et al., 2008; Gondolf, 2012). The positive results of many of these studies have led most of the states (44 according to Maiuro and Eberle, 2008) to adopt such programmes as part of the coordinated community response to DVA. Each state, however, has its own BIP program with varying screening, assessment, content, modality, and program length (Maiuro and Eberle, 2008, see also Gondolf, 2012).

In the UK, a BIP named Cautioning and Relationship Abuse (CARA) was initially trialed for first-time DVA offenders^1^ in Hampshire, in 2012–2014 (Strang et al., 2017). Findings were that 35% fewer men receiving the Service re-offended against their partner, compared to those in the control group, and further harm to victims was reduced by over a quarter. Project CARA (the “CARA Service”) is currently operating in Hampshire, West Midlands, Avon and Somerset, Dorset, Thames Valley, Northamptonshire and jointly in Norfolk and Cambridgeshire. The three original Ministry of Justice-led two-tier pilots (Leicestershire, Staffordshire, and West Yorkshire) also have dispensation to use CARA, but only for standard risk cases.^2^ Since 2019, the CARA Service has also been available for DVA offenders targeting other family members (non-intimate DVA, such as child-on-parent abuse).

Police responses to DVA are guided by a policy of positive action (College of Policing, 2020), and this generates a high volume of arrests for DVA incidents. Most of the cases (e.g., 55% study by Rowland, 2013) are disposed of by way of no further action (also see Jarman, 2011; Cornelius, 2013). Police and partner agencies have sought ways to influence offender behavior through education to prevent further harm to current or future victims. Conditional Cautions were introduced as a low-cost way of addressing this and to provide victims, who may not wish to attend court, with an alternative positive means of disposal. The attachment of conditions to a Caution presents an opportunity to tailor the law enforcement response to the needs and circumstances of each offence type. Correctly designed and delivered, the conditions could contribute to the overall goal of reducing that crime type. Accordingly, the CARA service was implemented as part of the Conditional Caution to help reduce total harm from DVA.

Project CARA is aimed at low-risk offenders. Their design is compatible with RNR (risk-need-responsivity) principles (see Andrews and Bonta, 2007). The Table 1 shows how CARA relates to the RNR principles.

**TABLE 1:** RNR principles vis-a-vis CARA.

RNR Principle (Hanson et al., 2017)	How does CARA design address RNR principle	What does CARA design do to address RNR principle	Evidence-base
Risk Principle: Match the intensity of services to a person’s level of risk for criminal activity	Accepts referrals based on risk level: standard ( = low) risk. Low risk = a chargeable offence. However, attending the CARA programme is a condition attached to an out of court disposal. OOCDs offer a way of dealing with low level and first time offending in a proportionate way i.e., not taking the offender to court (Gibbs, 2018).	Minimal intervention in line with low-risk offending: only 2 workshops; using a participatory/group-work approach.	Matching low criminogenic risk with low treatment intensity appears to optimize reduction in recidivism (Bonta et al., 2000). Of those cautioned in 2015, 15% reoffended within a year, compared to 30% of those who received a conditional or absolute discharge in court (Gibbs, 2018).
Need Principle: Target criminogenic needs (factors that contribute to the likelihood of new criminal activity)	Aims to help offender identify criminogenic attitude and move towards changing it. Aims to create offender insight into non-criminogenic needs and move towards addressing them.	Safe space to review the antecedents and consequences related to the offence: using motivational interviewing (MI) and cognitive behavioral therapy (CBT).	Addressing criminogenic needs is associated with an average 19% difference in recidivism between treated and non-treated cohorts (Andrews and Bonta, 2006). Using CBT produces an average difference in recidivism of 23% between treated and non-treated cohorts (Andrews and Bonta, 2006).
Responsivity Principle: Account for a person’s abilities and learning styles when designing services	Chronic or entrenched criminogenic and non-criminogenic needs (which challenge the individual’s ability to change their behaviors or environment) are met by onward self-referral made possible by the attitude change and insight (e.g., to DA perpetrator, debt, gambling, substance misuse and other programmes).	CARA workshop facilitators signpost/support onward referral to local programmes for offenders who have acquired insight and motivation to address criminogenic and non-criminogenic needs likely to promote recidivism.	Use of appropriate level of treatment for all three principles average recidivism difference is 35% when delivered in community settings (Andrews and Bonta, 2007).

In summary, Project CARA is an awareness-raising program that promotes behavior change for male and female individuals who are first-time DVA offenders. The intervention comprises two five-hour workshops that offenders attend as the core conditions of the Caution. The Service is delivered by teams of workshop facilitators from a third sector organization, the Hampton Trust. The victim informs the initial police-led risk assessment and decision to administer the Caution; and provides feedback on the offender’s progress/compliance between workshops. More details on CARA and a Theory of Change can be found in Christie et al. (2022).

In this study, we analyze the impact and economic benefit of CARA for two of the police force areas where it was introduced. Unlike previous studies, this study is based on data from two distinct UK police force areas with different socio-economic and demographic characteristics; the West Midlands Police Force and the Hampshire Constabulary. Modern machine learning techniques are used for the data analysis where we use matching methods to create control groups to assess the impact and economic benefit of CARA. The data contain information on offences that took place in the period between December 2018 and November 2019. The West Midlands Police provided us data on 539 offenders, including 195 recipients of CARA and 344 in the control group. The Hampshire Constabulary provided us with data on 549 individuals, 309 of which were in the control group, and 240 in the treatment group receiving CARA.^3^

We employed propensity score matching to make the CARA intervention group comparable to the control group based on observable characteristics. Propensity score matching (PSM) is a popular statistical method introduced by Rosenbaum and Rubin (1983), which removes the impact of confounding variables on self-selection into the treatment group. PSM is a quasi-experimental method which constructs an artificial control group by matching each treated unit with a non-treated unit of similar characteristics. The impact of the intervention is then calculated using these matches. The explanatory variables for the matching process were a host of individual characteristics that could affect the outcomes of interest. Five outcome indicators of treatment success were measured at two time periods, 6 and 12 months after the CARA referral date: a re-offence, a re-arrest, the number of re-offences and re-arrests, and the severity of crimes. An offence was designated a crime if it had been entered into the Police National Computer (PNC) system. To measure crime severity, the Cambridge Crime Harm Index (CHI) (Sherman, 2020) was used.

For West Midlands, in terms of the profile of offenders who attended the CARA Service, in comparison to the control group,^4^ they were more likely to be older (fewer 22–30 years) and White (with a notable disparity in the low number of Black offenders). Attendees were more likely to have: a history of alcohol misuse, a same-sex partner (marginally/control group had none), an older victim, and a White victim (with a notable disparity in the low number of Black victims). The offenders who attended the CARA Service were less likely to have a history of drug misuse. However, they were similar to the control group in terms of the likelihood of being unemployed, having a personality disorder, having a majority of female victims, be mentally ill, or have an ailment. For Hampshire, offenders shared similar characteristics to those of West Midlands, with the main differences being that a) there was very little non-White representation in both treatment and control groups, and b) the Hampshire control and treatment groups had higher recidivism than West Midlands ones.

Our analysis found that the CARA Service has a significant impact on recidivism. On average, the CARA Service reduced offences by 81% in the first six months and by 65% in the first 12 months for West Midlands. For Hampshire, CARA reduced offences by 39% in the first 6 months and by 41% in the first 12 months.

To calculate the economic benefits of the CARA service, the cost of the average crime was measured using the Heeks et al. (2018) Home Office report on the economic and social costs of crime (HOCC). The economic benefits analysis suggests that the economic benefits of introducing the CARA Service into a police force area are significant, even using conservative estimates. For West Midlands, the net benefit of the CARA Service was estimated to be £152,775.94 in a period of 6 months and £195,729.09 annually. For Hampshire, the net benefit is estimated to be £596,066 in the first 6 months and £780,864.40 annually. For West Midlands, the benefit-cost ratio is equal to 2.75, meaning that for each pound invested in a CARA project 2.75 pounds are gained. For Hampshire, the benefit-cost ratio is equal to 11.10, meaning that for each pound invested in a CARA project 11.10 pounds are gained. These numbers are conservative estimates of the true impact of the CARA effect and the actual benefit of CARA could be greater.

The paper is organized as follows. Section “2. Materials and methods” describes the measures used and the PSM methodology. Section “3. Results” provides the descriptive statistics of the data, the statistical analysis, and the impact evaluation of CARA. Section “4. Economic benefits” presents the cost-benefit analysis. Section “5. Conclusion” concludes the paper.

## 2. Materials and methods

The West Midlands Police and Hampshire Constabulary datasets differ in the set of available variables and therefore we analyze them separately to take advantage of the extra information where available, and to avoid data pooling assumptions. This study is unique in that the impact of the intervention is examined across two independent samples. It is also unique in that it uses two different crime severity measures.

The following measures were used in the analysis. First, all individuals have been risk-assessed based on the Domestic Abuse, Stalking and Harassment (DASH) Risk Identification and Assessment and Management Model (DASH, 2009). DASH was implemented across all police services in the UK from March 2009, having been accredited by the ACPO Council, now known as theNational Police Chief Council (NPCC). All police services and a large number of partner agencies across the UK use this common checklist for identifying, assessing and managing risk. The DASH index classifies individuals into one of three categories: standard risk, medium risk and high risk.

To measure the severity of crimes, we employ first the Cambridge Crime Harm Index (CHI, see Sherman, 2020). According to the CHI, the harm score for a crime is the default prison sentence that an offender would receive for committing it, if the crime was committed by a single offender with no prior convictions. For minor crimes that would instead result in a fine, the harm score is the number of days it would take someone with a minimum wage job to earn the money to pay the fine.

The second crime severity index is the Crime Severity Score index (CSS) taken from the ONS (Office for National Statistics, 2022b). The CSS is a weighted index that reflects the relative harm of an offence to society and the likely demands on the police.

The collected police data are not the outcome of a randomized control trial, which means we need to exercise *ex post* statistical control for confounding variables that affect both the treatment group formation (i.e., probability of selection into the treatment group) and the treatment group outcomes. To illustrate this point, suppose such a variable is age; older people may be more likely to participate in the CARA treatment, and CARA recipients may show reduced recidivism only because older individuals have relatively lower re-offending rates rather than from any effect of CARA.

We use propensity score matching, a commonly used statistical technique (see, Rosenbaum and Rubin, 1983), to remove the effects that arise from confounding variables. This method is a quasi-experimental method that seeks to mimic randomization to overcome issues of selection bias that plague non-experimental settings. This method will allow us to provide a valid estimate of the intervention effect. The economic impact of the change in outcomes between the treatment and control group is based on this estimated effect and the calculations appear in section “3. Results.”

Operationally, PSM involves a three-step process.

• Step 1: A logistic regression is estimated where the dependent variable is a dummy (binary) variable that takes the value one if an individual received the intervention and the value zero otherwise. The explanatory variables are individual characteristics, considered as confounding factors affecting the treatment sample and the treatment outcome.
• Step 2: The predicted probabilities from the logistic regression in step 1 are used to create “matching pairs” of “similar” individuals. In each pair, one individual will have received the treatment, and one will not.
• Step 3: The average difference in recidivism over all such pairs constitutes a reasonable estimate of the average treatment effect (i.e., the impact of CARA).

Propensity score matching (PSM) matches individuals in the control and treatment groups. Because these individuals are the same (or almost identical) according to a set of observed characteristics, any differences in re-offending are attributed to the treatment. The matching is done by comparing the probability that an individual is assigned to the treatment group. Therefore, a matched pair comprises two individuals with the same probability of being assigned to the treatment group, yet one of them belongs to the control group. This probability is called the propensity score. The first step of PSM is to use logistic regression to calculate the propensity scores of each individual in the sample, the second step is to match these individuals according to their propensity scores. As the number of individuals in the control group is different from those in the treatment group, we use kernel-based matching. We selected the Epanechnikov kernel with a bandwidth of 0.06, which are common choices in the literature, see, e.g., Heckman et al. (1997). PSM is standard in this literature, see e.g., Cox and Rivolta (2021).

## 3. Results

### 3.1. Descriptive statistics: West midlands police data

In this section, we present West Midlands Police (WMP) dataset and its analysis. This is the richer dataset of the two, in terms of observable perpetrator characteristics. As stated above, there are 539 offenders, including 195 recipients of CARA, and 344 in the control group. This information is shown in Tables 2 and 3. In addition, four eligible offenders breached their conditional caution by not attending any of the CARA workshops. Given that their number is too small to form a separate study group, and given that they did not receive the treatment, we allocated them to the control group leading us to a final sample of 191 recipients of CARA and 348 in the control group. The inclusion of these four cases on the control group does not cause bias as the analysis is not based on this sample but on the balanced sample created by PSM.^5^ The data show that all CARA participants have been through the full 10-hour duration of the intervention.

**TABLE 2:** West Midlands Police Data Descriptive Statistics - Means of general offender characteristics.

Variable	Control group	CARA group	Equality of means or proportions test (*p*-value)
Size	348	191	–
Gender - Female	9.48%	7.85%	0.525
Age	34.24 (10.90)	38.11 (11.89)	0.000
White	69.82%	77.48%	0.056
Black	11.2%	7.32%	0.148
Asian	18.96%	15.18%	0.270
Unemployed	23.28%	23.04%	0.949
Mental health issues	16.09%	18.32%	0.508
Personality disorder	19.54%	23.04%	0.338
History of alcohol misuse	1.72%	5.24%	0.021
History of drug misuse	7.18%	3.14%	0.053
Ailment	14.37%	16.23%	0.562
Same-sex partner	0%	2.09%	0.006
DV arrests in the past year	0.066 (0.260)	0.052 (0.285)	0.571
Offences in the past year	0.57%	0%	0.293
CSS index for offences in the past year	0.091 (0.528)	0.535 (4.65)	0.079

The standard deviation for continuous variables appears in the parenthesis next to the mean.

**TABLE 3:** West Midlands Police Data Descriptive Statistics - Means of offender crime characteristics.

Variable	Control group	CARA group	Equality of means or proportions test (*p*-value)
CARA offence DASH-Stand	36.78%	42.24%	0.199
CARA offence DASH-Med	56.03%	54.45%	0.723
CARA offence DASH-High	5.17%	2.09%	0.084
CARA offence alcohol misuse	26.15%	30.89%	0.240
CARA offence victim is female	90.80%	92.15%	0.597
CARA offence victim’s age	32.68 (10.725)	34.54 (11.208)	0.059
CARA offence victim is White	72.41%	80,1%	0.048
CARA Offence CSS score	9.258 (40.859)	13.437 (54.74)	0.316
Reoffended within 6 months	6.90%	2.09%	0.016
Reoffended within 12 months	10.06%	5.24%	0.052
Rearrested within 6 months	15.80%	8.90%	0.024
Rearrested within 12 months	21.84%	15.71%	0.086
Number of re-offences after 6 months	0.140 (0.644)	0.026 (0.19)	0.016
Number of re-offences after 12 months	0.206 (0.761)	0.068 (0.308)	0.016
Number of re-arrests after 6 months	0.238 (0.702)	0.099 (0.333)	0.010
Number of re-arrests after 12 months	0.367 (0.943)	0.172 (0.418)	0.006
Cambridge Crime Harm Index after 6 months	3.265 (27.526)	0.916 (6.904)	0.247
Cambridge Crime Harm Index after 12 months	3.946 (27.971)	1.293 (7.240)	0.198

The standard deviation for continuous variables appears in the parenthesis next to the mean.

The WMP control group is made up of individuals who received a caution, community resolution, or were NFA’d (No Further Action) to being charged, during the time when CARA was not available (from December 2016 to May 2017), or who were part of the control group in a WMP pilot study that took place between June 2017 and November 2018. Finally, there are a few individuals from December 2018-onwards who slipped through the net and were not offered CARA, even though it was available.

We examine five measures of treatment success across two time periods:

(i) If an individual reoffends,
(ii) If an individual is re-arrested,
(iii) The number of offences post-CARA,
(iv) The number of arrests post-CARA,
(v) The severity of crimes post-CARA.

We examine these variables at the end of 6 and 12 months after the CARA referral date. In the above, an offence is a crime that has been entered into the Police National Computer (PNC) system.

We now present some descriptive statistics for the offender characteristics. The variables in the dataset are: Table 4

**TABLE 4:** Offender characteristics and CARA crime.

• Offender:	• CARA crime:
- age - gender - ethnicity - employment - mental health status - personality disorders - alcohol misuse - drug misuse - ailments - same-sex partners - prior DV arrests (1 year) - prior offences (1 year)	- severity of prior offences - prior arrests (1 year) severity - risk assessment - alcohol involvement - victim’s gender - victim’s age - victim’s ethnicity

In the above, we refer to the CARA crime as the incident based on which an offender is considered for CARA treatment. For the control group, there is a corresponding incident of the same level, based on which the control group was created (although CARA was never offered).

#### 3.1.1. Offender and victim profiles

The aim of this section is to present an overview of the sample and not to arrive at a conclusion about the similarity between the treatment and the control group. The confounding variables of self-selection will be addressed by PSM in sections “3.3. PSM for West Midlands Police data” and “3.4. PSM for Hampshire constabulary” below. Tables 2, 3 present some descriptive statistics for the variables in the WMP sample. The third column of both tables contains the *p*-values for tests of statistically significant differences in the means or the proportions between the treatment and control groups. These *p*-values are also reported in the parentheses in the text below. Table 2 includes general offender characteristics while Table 3 specializes on crime and recidivism. The offender gender is dominated by males in both control and treatment groups. The “mental health” dummy variable captures common mental health issues such as anxiety, depression, and attention deficit hyperactivity disorder. Suicidal and self-harm cases appear in the dummy variable “personality disorders.” The “alcohol misuse” dummy variable includes both alcohol dependence and alcoholism. The dummy variable “ailment” consists of a range of health issues, from asthma to lack of body organs. The “same-sex partner” dummy variable captures whether the offender and the victim are of the same sex. The control and treatment groups are similar in proportions of females (*p* = 0.525), mental health issues (0.508), personality disorders (*p* = 0.338), ailments (*p* = 0.562), and unemployment (*p* = 0.949). Alcohol misuse (*p* = 0.021), drug misuse (*p* = 0.053), and same sex partners (*p* = 0.006) were greater in the treatment group. The participants in the CARA treatment are on average older than those in the control group (*p* < 0.001) and there is a higher percentage of White ethnicity (*p* = 0.056).

Individuals in the treatment group have not been charged before the CARA offence, unlike some in the control group, although their difference is statistically non-significant (*p* = 0.293). The two groups are similar in numbers of prior domestic violence related arrests (*p* = 0.571), although there is a marginally higher CSS score for the treatment groups’ past offences (*p* = 0.079). The CSS of the treatment group for past crimes comes from the arrest data; these individuals were not charged for these crimes.

#### 3.1.2. CARA offence and re-offending

Table 3 presents statistics related to the CARA offence and future recidivism. Looking at the characteristics of the CARA offence (or the corresponding control group offence), we break the DASH index into three binary variables: standard risk, medium risk, and high risk, and then compare proportions across the two groups. The treatment and control groups are similar in proportions of individuals in the standard (*p* = 0.199) and medium (*p* = 0.723) risk categories, but there is some slight discrepancy in the high risk (*p* = 0.084) category towards the control group.^6^ The alcohol involvement at the CARA offence was similar in the two groups (*p* = 0.240) and so is the percentage of female victims (*p* = 0.597) and the average CSS score (*p* = 0.316). However, the CARA group has victims of higher average age (*p* = 0.059), and which are more ethnically White (*p* = 0.048).

We now move on to the recidivism variables, which are used to measure the effect of CARA. In terms of the percentage of individuals who reoffended within a period of 6 months, unconditionally, there is a large difference between the treatment and control groups (*p* = 0.016) with the control group displaying higher recidivism. The difference is smaller after 12 months but still statistically significant (*p* = 0.052). A similar picture can be seen in terms of individuals who were re-arrested after 6 and 12 months (*p* = 0.024 and *p* = 0.086, respectively). The number of re-offences and the number of re-arrests for both the 6- and 12-month periods is higher for the control group (re-offences: *p* = 0.016 and *p* = 0.016 for 6 and 12 months, respectively; re-arrests: *p* = 0.010 and *p* = 0.006 for 6 and 12 months, respectively), with all differences being statistically significant. The CHI index is also higher for the control group for both periods, although the differences to the treatment group are not statistically significant (*p* = 0.247 and *p* = 0.198).

### 3.2. Descriptive statistics: Hampshire constabulary data

The Hampshire Constabulary (HSC) dataset has a similar size to that of WMP and contains similar information, which allowed us to use the same methods for analysis. The main differences between the two datasets are that the HSC has less data on victim characteristics, mental health issues, alcohol misuse, and employment. Furthermore, another difference is that we have data on the number of offences at arrest but not the number of offences which have been entered in the PNC. Given that the number of offences at arrest is equal to or more than the number of offences that have been charged, we expect that this measure will lead to slightly less conservative estimates of the impact of CARA when compared to the WMP data. However, the difference should only be marginal. As can be seen from the WMP dataset, in which the number of offences at arrest and the number of offences in the PNC are documented, the two numbers coincide for 504 out of 539 individuals, and there is an extra offence at arrest for another 23 individuals. Finally, the severity index used is not the CHI as in WMP, but the CSS. The new index allows us to examine the robustness of the previous findings.

Table 5 presents offender demographic statistics for the HSC sample. The first row shows that there are 309 offenders in the control group and 240 in the treatment group, making up a sample of 549 individuals. Like the WMP dataset, most perpetrators are male with equal proportions amongst the two groups (*p* = 0.918). The treatment and control groups are similar in terms of mental health issues (*p* = 0.142), proportion of White individuals (*p* = 0.709), and domestic-violence related arrests in the past year (*p* = 0.764). There is a higher drug misuse in the control group (*p* = 0.015), the participants in the CARA service are older (*p* < 0.001), and the CSS index for past offences is higher for the control group (*p* = 0.055).

**TABLE 5:** Hampshire Constabulary Data Descriptive Statistics - Means of General offender characteristics.

Variable	Control group	CARA group	Equality of means or proportions test (*p*-value)
Size	309	240	–
Gender - Female	19.09%	18.75%	0.918
Mental health issues	9.71%	6.25%	0.142
History of drug misuse	12.94%	6.67%	0.015
Age	31.60 (11.528)	35.57 (11.839)	0.000
White	34.30%	35.83%	0.709
DV arrests in the past year	0.200 (0.551)	0.187 (0.45)	0.764
CSS index for offences in the past year	116.93 (372.5)	64.63 (226.1)	0.055

The standard deviation for continuous variables appears in the parenthesis next to the mean.

The first three rows of Table 6 refer to the DASH risk in the sample. There are more standard risk individuals in the control group (*p* = 0.030), the two groups have a similar number of medium risk individuals (*p* = 0.434) and there are more high-risk individuals in the treatment group (*p* = 0.013).

**TABLE 6:** Hampshire constabulary data descriptive statistics–Means of offender crime characteristics.

Variable	Control group	CARA group	Equality of means or proportions test (*p*-value)
CARA offence DASH-Stand	43.68%	34.58%	0.030
CARA offence DASH-Med	50.8%	54.16%	0.434
CARA offence DASH-High	5.5%	11.25%	0.013
CARA offence alcohol misuse	28.48%	32.50%	0.308
CARA Offence CSS score	92.95 (149.48)	116.44 (176.4)	0.092
Reoffended within 6 months	40.78%	27.92%	0.001
Reoffended within 12 months	44.01%	29.17%	0.000
Rearrested within 6 months	10.36%	13.33%	0.280
Rearrested within 12 months	10.68%	14.17%	0.215
Number of re-offences after 6 months	0.867 (1.649)	0.379 (0.698)	0.000
Number of re-offences after 12 months	1.187 (2.477)	0.462 (0.909)	0.000
Number of re-arrests after 6 months	0.135 (0.47)	0.154 (0.425)	0.638
Number of re-arrests after 12 months	0.139 (0.473)	0.170 (0.475)	0.437
CSS Index after 6 months	100.78 (421.7)	61.51 (348.99)	0.244
CSS Index after 12 months	146.47 (524.5)	66.9 (352.02)	0.043

The standard deviation for continuous variables appears in the parenthesis next to the mean.

The ratio of people with alcohol use in the CARA crime (or the corresponding crime for the control group) between the control and treatment groups is similar (*p* = 0.302), and so is the CSS score (*p* = 0.092).

Moving on to the recidivism variables, we observe that in terms of the percentage of individuals who reoffended within a period of 6 months, unconditionally, there is a large difference between the treatment and control groups (*p* = 0.001) with the control group displaying higher recidivism. The difference is slightly larger after 12 months (*p* < 0.001). However, there seems to be no difference in terms of individuals who were re-arrested after 6 and 12 months (*p* = 0.280 and *p* = 0.215, respectively).^7^ The number of re-offences both the 6- and 12-month periods is higher for the control group (*p* < 0.001 and *p* < 0.001, respectively), while the number of arrests is similar for both periods (*p* = 0.638 and *p* = 0.437 for 6 and 12 months, respectively). The CSS index is also higher for the control group for both periods, although the difference becomes statistically significant after 12 months (*p* = 0.043).

### 3.3. PSM for West Midlands Police data

The set of characteristics based on which individuals are matched may impact the matching and the estimation of the average treatment effect. Therefore, we employ two sets of characteristics (information sets), based on the variable set described above, to ensure that we capture all the confounding variables. Furthermore, a third set of matching characteristics is created using a machine-learning method called backwards stepwise regression, which identifies the most important variables determining treatment selection. This method can also shed light on the characteristics that make specific individuals ineligible for CARA and may call for CARA policy changes. The two information sets are presented in Tables 7, 8.

**TABLE 7:** Propensity score matching basic information sets (West Midlands Police).

Basic information set	Full information set
Age, gender, ethnicity, unemployment, alcohol misuse, mental health, past severity (1 year), past arrests (1 year), CARA severity, CARA risk.	Age, gender, ethnicity, unemployment, alcohol misuse, drug misuse, mental health, ailment, personality disorder, past severity (1 year), past arrests (1 year), CARA severity, CARA risk (DASH standard, DASH medium, DASH high), CARA alcohol involvement, gender of the victim, ethnicity of victim, age of the victim

**TABLE 8:** Propensity score matching machine learning information set (West Midlands Police).

Machine learning information Set at 10% sig	Machine learning information Set at 20% sig
Age (***, +), Victim’s age (**,−) Victim is white (*, +) Alcohol misuse (*, +)	Age (***, +), Victim’s age (**,−) Victim is white (**, +) Alcohol misuse (*, +) Drug misuse (−)

*Refers to *p* < 0.10, **refers to *p* < 0.05, ***refers to *p* < 0.01.

In the above information sets, we do not include the variables, same-sex partnership, and past offences because we do not have data on these for both the treatment and the control group.

The machine learning method begins with the full information set, which contains 20 regressors and removes the variables that are not statistically significant in a stepwise manner, starting with the least non-significant. For robustness, we examine two versions of the algorithm; the first version drops variables that are not significant at the 10% level. The second version drops variables that are not significant at the 15% level. The latter higher significance level is a more conservative view of the factors affecting the probability that someone receives treatment. We denote each variable’s statistical significance at the 1% (***), 5% (**), and 10% (*). Next to the significance, we also report the sign of the variable’s coefficient in the regression. The interpretation is as follows. A positive sign means that, as the variable increases, it increases the probability of CARA participation; a negative sign means that, as the variable increases, the probability of CARA participation drops.

At both the 10% and 15% stepwise regression levels, the most important variables determining CARA participation are the offender’s age, the victim’s age, if the victim is White, if the offender has alcohol-related issues, and secondarily if the offender has drug-misuse issues. The older the offender is, the higher the probability that they will be admitted to CARA. On the other hand, the older the victim is, the lower the probability that the offender will be admitted to CARA. The likelihood of joining CARA also increases if the victim is White. Finally, alcohol misuse increases the likelihood of being administered with the treatment, while drug misuse reduces it. For the remaining analysis (matching), we maintain the 10% significance machine learning information set as drug misuse does not significantly impact the results.

The average treatment effects calculated after matching are presented in Table 9 below. The table shows results for the ten outcome variables and the three information sets described earlier. All results in the “Diff” columns are negative, showing evidence across the board that CARA reduces recidivism, no matter how it is measured. The results are statistically significant almost everywhere regarding re-offences and re-arrests but non-significant in terms of CHI reduction. The results vary across the three information sets but only a little; this is strong evidence of the validity of the machine learning information set. When we examine the impact of CARA in the 6 months after the referral, we can see significant reductions in recidivism. Based on the machine learning information set, the number of re-offenders is reduced by 70%, while the number of individuals re-arrested is reduced by 39%. The number of re-offences is reduced by 81%, while the number of re-arrests is reduced by 56%. Finally, the Crime severity score index of crimes is reduced by 70%. When we examine the impact of CARA after 12 months, we observe smaller treatment effects. The number of re-offenders is reduced by 43%, while the number of individuals re-arrested is reduced by 25%. The number of re-offences is reduced by 65%, while the number of re-arrests by 52%. Finally, the Crime severity score index of crimes is reduced by 62%. Statistical significances are also weaker after 12 months.

**TABLE 9:** Propensity score matching results (West Midlands Police).

	Basic information set						Full information set							Machine learning information set (10%)							
Variables	Tr	Con	Diff	SE	t-stat	%Ch	B	Tr	Con	Diff	SE	t-stat	%Ch	B	Tr	Con	Diff	SE	t-stat	%Ch	B
Re-offending 6 months	0.016	0.060	−0.044	0.017	−2.50***	−73%	1.85	0.021	0.063	−0.041	0.019	−2.17**	−65%	1.55	0.021	0.069	−0.048	0.018	−2.62***	−70%	1.6
Re-arrested 6 months	0.086	0.141	−0.055	0.030	−1.84*	−39%	1.3	0.085	0.137	−0.052	0.030	−1.72*	−38%	1.25	0.089	0.145	−0.055	0.030	−1.84*	−39%	1.3
Number of re-offences 6 months	0.021	0.123	−0.102	0.041	−2.49***	−83%	5	0.026	0.137	−0.110	0.043	−2.55***	−80%	5	0.026	0.148	−0.122	0.041	−2.96***	−81%	5
Number of re-arrests 6 months	0.096	0.208	−0.111	0.048	−2.29**	−53%	3	0.095	0.215	−0.119	0.050	−2.35***	−55%	3	0.100	0.226	−0.125	0.048	−2.58***	−56%	3
CHI 6 months	0.688	2.86	−2.17	1.72	−1.26	−76%	5	0.904	2.95	−2.04	1.83	−1.12	−69%	5	0.925	3.07	−2.15	1.73	−1.24**	−70%	5
Re-offending 12 months	0.043	0.083	−0.040	0.023	−1.74*	−48%	1.4	0.047	0.091	−0.043	0.024	−1.76*	−47%	1.3	0.052	0.092	−0.040	0.024	−1.64*	−43%	1.2
Re-arrested 12 months	0.155	0.205	−0.049	0.036	−1.36	−24%	1.1	0.154	0.215	−0.061	0.037	−1.65*	−28%	1.1	0.158	0.212	−0.053	0.036	−1.48	−25%	1.1
Number of re-offences 12 months	0.059	0.173	−0.114	0.050	−2.25**	−66%	5	0.063	0.193	−0.129	0.053	−2.43***	−67%	5	0.068	0.196	−0.127	0.051	−2.50***	−65%	5
Number of re-arrests 12 months	0.166	0.339	−0.172	0.064	−2.69***	−51%	2	0.164	0.371	−0.206	0.066	−3.09***	−56%	2	0.174	0.360	−0.186	0.064	−2.89***	−52%	2
CHI 12 months	1.07	3.39	−2.31	1.75	−1.32	−68%	5	1.28	3.44	−2.16	1.86	−1.16	−63%	5	1.30	3.49	−2.18	1.76	−1.24	−62%	4

*Refers to *p* < 0.10, **refers to *p* < 0.05, ***refers to *p* < 0.01. “Tr”, reports the average output variable among treated subjects in the matched sample. “Con”, reports the average output variable among control group subjects in the matched sample. “Diff”, reports the difference between the previous two averages, which constitutes the average treatment effect on the treated. “S.E.”, reports the standard error of the difference. “*t*-stat”, reports the *t*-statistic. “%Ch”, reports the percentage change in the average output variable because of the treatment. “B”, reports the outcomes of the bounds robustness check of Rosenbaum (2002) which tells us that the results are unlikely to be affected by the presence of unobservable factors.

To summarize the above findings, CARA has a significant impact on recidivism, particularly in the first 6 months. The effect is substantial also after 12 months but is less pronounced. A notable finding is that, while the reductions in re-offenders and numbers of re-arrests and re-offences are generally statistically significant, this is not the case with CHI. This result contrasts with Strang et al. (2017) who document such a reduction. The result may be driven by the significantly lesser (less harmful) offences found in the West Midlands Police sample; the average CHI is 6.32, which is 25% to 45% smaller than the 8 to 11 CHI averages found in Strang et al. (2017).

We conclude this section with a few words on the robustness and statistical validity of the methodology. First, it is encouraging to see that the results are stable across the information sets, indicating that there is likely no confounding variable problem. Second, in all the above regressions, the balanced (matched) samples do not have any statistically significant differences in the means of the variables of the information sets. Third, only 1–5 observations in each regression are outside the common support. These results are unreported for brevity but are available upon request.

Finally, the seventh column of each information set in Table 9 reports the ratio of the odds of receiving treatment for two matched individuals i and j with different unobserved characteristics. This ratio can indicate how sensitive the above results are to potential unobservable confounding variables. Consider the full information set results on the number of re-offenders after six months. We find that, for the assumption that the treatment effect in our sample is overestimated (in absolute terms) to get rejected, the unobservable confounding factor [B value (Rosenbaum, 2002)] would have to increase the odds of receiving treatment by 1.55 times. For the reduction of the number of re-offences to be overestimated, the confounding factor would have to increase the odds of receiving treatment by at least five times (given that this is the maximum allowed). Therefore, if the results are susceptible to confounding factors, these factors need to have a dramatic effect in order to cast doubt on our results, which we do not think is likely.

As a last robustness check, we examined carefully whether the above results are originating from one of the DASH risk categories. Unfortunately, the available sample does not provide enough power for this analysis. Splitting the sample between standard and medium risk cases creates two smaller subsamples with 209 observations for the standard risk sample and 299 observations for the medium risk sample. These sample sizes include both the control and treatment group and lead to statistically non-significant results.

### 3.4. PSM for Hampshire constabulary

We now apply the propensity score matching methodology that has been used previously in the WMP data. Because we have fewer variables, we only employ one information set which consists of all the variables at our disposal, as they were presented in the previous section.

Table 10 shows that CARA has had a significant effect on the reduction of re-offences. There was a drop in re-offending probability by 21% within 6 months and 23% within 12 months. The number of re-offences is reduced by 39% within 6 months and by 41% within 12 months. We can see that CARA has a statistically significant effect. The results are robust to confounding variables with a B value of three. Comparing these to the WMP data, the reductions caused by CARA are qualitatively similar. In terms of the magnitude of reduction, the Hampshire Constabulary data show that CARA has about half the effect of the West Midlands Police data. However, the CARA effect does not drop in strength over the 12-month period, unlike WMP. The difference between the two can be explained by comparing the statistics in Tables 2, 6 for the re-offending probabilities and for the number of re-offences. Clearly, perpetrators in the Hampshire Constabulary seem more prone to recidivism which may be due to area idiosyncratic characteristics. CARA has a smaller effect in magnitude but is more long-lasting when compared to WMP. However, in both areas, CARA’s effect is statistically significant.

**TABLE 10:** Propensity score matching results (Hampshire Constabulary).

	Basic information set						
Variables	Tr	Con	Diff	SE	t-stat	%Ch	B
Re-offending 6 months	0.280	0.355	−0.074	0.043	−1.74*	−21%	1.35
Re-arrested 6 months	0.133	0.098	0.035	0.029	1.19	36%	1.05
Number of re-offences 6 months	0.380	0.623	−0.245	0.095	−2.55***	−39%	3
Number of re-arrests 6 months	0.154	0.123	0.031	0.041	0.76	25%	1
CSS 6 months	61.774	68.361	−6.587	35.550	−0.19	−9%	5
Re-offending 12 months	0.292	0.380	−0.088	0.043	−2.03**	−23%	1
Re-arrested 12 months	0.142	0.099	0.042	0.030	1.42	42%	1
Number of re-offences 12 months	0.464	0.795	−0.331	0.125	−2.64***	−41%	3
Number of re-arrests 12 months	0.171	0.124	0.047	0.043	1.09	38%	1
CSS 12 months	67.179	97.646	−30.466	40.520	−0.75	−31%	5

*Refers to *p* < 0.10, **refers to *p* < 0.05, ***refers to *p* < 0.01. “Tr”, reports the average output variable among treated subjects in the matched sample. “Con”, reports the average output variable among control group subjects in the matched sample. “Diff”, reports the difference between the previous two averages, which constitutes the average treatment effect on the treated. “S.E.”, reports the standard error of the difference. “*t*-stat”, reports the *t*-statistic. “%Ch”, reports the percentage change in the average output variable because of the treatment. “B”, reports the outcomes of the bounds robustness check of Rosenbaum (2002) which tells us that the results are unlikely to be affected by the presence of unobservable factors.

Looking at re-arrests, the estimations show that individuals from the treatment group are as likely to get re-arrested as those from the control group. There is no statistically significant difference between the two. These results are somewhat different from the WMP findings where the reduction in arrests was statistically significant. The insignificance may be driven by omitted variables that may affect sample selection. An indicator supporting this claim is the value of the bounds robustness check B, which is equal to, or very close to one. Such variables could be the victim characteristics or perpetrator alcohol misuse; these variables were available in the WMP dataset, where B was quite high.

Finally, when it comes to the CSS index, we estimate a reduction due to CARA, but once more the results are not significant. This is in line with the CHI results in the WMP dataset which were also not significant. This is further evidence that the results are robust to the measurement of crime harm. Overall, the results of this second dataset are in line with the findings from the WMP dataset in terms of re-offences and in terms of crime severity but not in terms of re-arrests.

## 4. Economic benefits

### 4.1. Economic benefits for WMP data

Table 11 provides a description and frequency of crimes in the sample. Notice that some offenders commit more than one crime at the same time, which leads to a number of crimes greater than the number of offenders. The third column includes the CHI employed as a measure of success in Strang et al. (2017) and our analysis, as explained below. The (weighted) average CHI in our sample is 6.32 days which is smaller than the 8 to 11 days averages found in Strang et al. (2017). Finally, the last two columns include the cost of crime classification and estimates from the Heeks et al. (2018) HOCC. These estimates are comprehensive and include estimates of the costs in anticipation of crimes, for example, burglar alarms, costs because of crime, for example, the cost of stolen or damaged property, and costs in response to crime, for example, costs to the police and criminal justice system. These estimates have been translated into 2020 prices using the Bank of England’s inflation calculator.

**TABLE 11:** Type and frequency of crimes (West Midlands Police).

Crime	Frequency	CHI (prison days)	HOCC classification	HOCC (GBP)
Common assault	115	1	Violence without injury	5,930
Assault occasioning ABH	128	10	Violence with injury	14,050
Battery	88	1	Violence without injury	5,930
Criminal damage under 5000	102	1	Criminal damage-other	1,350
Harassment without violence	75	10		0
Send communication/article conveying a threatening	40	2		0
Breach of a non-molestation order	8	5		0
Disclose private sexual photographs and films	6	5	Other sexual offences	6,520
Burglary residential	4	19	Domestic burglary	5,930
Grievous Bodily Harm	10	19	Violence with injury	14,050
Cannabis possession	10	2		0
Driving under the influence of alcohol	2	2		0
Threats to kill	9	10		0
Ill-treatment of child	4	5		0
Arson endangering life	2	365	Criminal damage-arson	8,420
Racially aggravated harassment-words	1	10		0
Stalking involving serious alarm	1	252		0
Harassment–In fear of violence	5	5		0
Threaten to damage property	2	2		0
Theft from dwelling	2	2	Domestic burglary	5,930
Coercive behavior	1	10		0
Resisting a constable	1	1		0
Criminal damage over 5000	6	2		0

To calculate the economic benefits of CARA, we start with the control group data and then apply the estimated CARA reductions, as these were estimated by the full information set and presented in Table 12. We measure the cost of crime using the Heeks et al. (2018) HOCC. The estimates in HOCC consider three main cost areas: the costs in anticipation of crime; the costs as a consequence of crime; and finally, the costs in response to crime. This index includes a wide range of costs, such as productivity loss, personal injury hospital admission costs, mental health costs, and police and criminal justice system costs. One of the limitations of HOCC is that these costs are calculated only for a short list of crimes, most of which are not found in our sample. Therefore, under this approach, we will only apply an economic cost to the most severe crimes in the sample. Consequently, we will underestimate the actual cost of crime.^8^

**TABLE 12:** Economic benefits of the CARA service (West Midlands Police).

		Number of crimes	Total cost of crime
Control Group	6 months	49	£296,019.68
	12 months	72	£434,967.69
Estimated costs after CARA	6 months	9.31	£56,243.74
	12 months	25.2	£152,238.74
CARA Benefit	6 months	39.69	£239,775.94
	12 months	46.8	£282,729.09

When calculating the economic benefits of CARA, we will only consider the reductions in re-offences and not the decreases in re-arrests or CHI. Concerning arrests, we will miss out on the costs of arrests that did not lead to an offence. These arrests have significant economic costs, and we do not have precise information on the cost of an arrest to the police. All of these suggest that the benefits we are presenting are underestimates. Concerning the reduction in the severity of the crime, we did not find any statistically significant changes; in other words, the crimes after the treatment are at the same level of severity as the crimes before the treatment.

The first step is to calculate the cost of the average crime in the sample, according to the HOCC index. The calculation is done using the data from Table 11. The cost of (weighted) average crime is given by multiplying the HOCC cost of the crime with its sample weight, given by the ratio of the frequency of the crime to the total number of crimes. We find that the cost of the average crime is £6,041.22. This cost is based on the finding that CARA does not affect crime severity, and therefore both control and treatment group crimes are used in the estimation. For robustness, we also calculate the average cost of crime using only control group data. Indeed, in the absence of CARA, the cost of the average crime would come from the control group only. This cost is estimated to be £6,034.23. This is another indication that CARA does not reduce the severity of crime. We proceed with our analysis by keeping the value £6,041.22 as the cost of the average crime, because it is estimated from a larger sample.

In the control group, we have 49 offences in the 6 months and 72 offences in the 12 months. According to the last column of Table 9, which contains the results of the machine learning information set, CARA on average reduces the offences by 81% in the 6 months and 65% in the 12 months. The economic benefit calculations appear in Table 12 below.

Consider the third column in Table 12. First, it contains the number of crimes/offences in the control group. Below that, it contains the predicted offences that we would have if the CARA treatment was applied to this control group. Then, further below, it reports the GBP amount of reduction, which is equal to the reduction in the number of crimes caused by CARA times the cost of each crime. For example, at the six-month interval, CARA is predicted to reduce the 49 crimes in the control group to only 9.31 crimes, a reduction of 39.69 crimes. Therefore, the economic benefit of CARA is that it prevents 39.69 crimes, each of which costs £6,041.22. Therefore, the economic benefit of CARA is 39.69 × £6,041.22 = £239,775.94.

However, the calculations above do not include the cost of CARA, which will have to be applied to all 348 individuals in the control group. To calculate the economic benefits above, we need to subtract the cost of CARA per individual. This cost is £250 per individual, in 2020, according to the Hampton Trust. It includes practitioner costs, supervision costs, management costs, administration costs, other costs, venue hire for two days, refreshments, practitioner travel expenses, IT, stationery, and organizational overheads (e.g., memberships, insurances, quality standards etc.). This cost estimate is based on an average group of 10 individuals in each workshop. In the control group, there are 348 individuals and the cost to put them through CARA would be £348 × £250 = £87,000. Therefore, the net benefit of CARA would be £239,775.94−£87,000 = £152,775.94 in a period of 6 months and £195,729.09 annually. The benefit-cost ratio is equal to £239,775.94/£87,000 = 2.75, meaning that for each pound invested in CARA 2.75 pounds are gained. The benefit-cost ratio is almost the same if we use the annual data.

### 4.2. Economic benefits for HSC data

We now turn to the economic benefits of CARA, as estimated from the HSC sample. Crime frequencies and costs are displayed in Table 13. This table corresponds to Table 11 in the WMP data. The economic analysis is the same as before. We find that the cost of the average crime is £5,702.89, strikingly close to the estimated cost of £6,041.22 in the WMP data.

**TABLE 13:** Crime frequency and cost of crime (Hampshire Constabulary).

Crime	Frequency	HOCC classification	HOCC (GBP)
Common assault	249	Violence without injury	5,930
Assault occasioning ABH	70	Violence with injury	14,050
Battery	42	Violence without injury	5,930
Criminal damage under 5000	133	Criminal damage-other	1,350
Harassment without violence	35		0
Send communication/article conveying a threatening	26		0
Breach of a non-molestation order	2		0
Disclose private sexual photographs and films	3	Other sexual offences	6,520
Grievous Bodily Harm	1	Violence with injury	14,050
Cannabis possession	1		0
Ill-treatment of child	4		0
Arson endangering life	1	Criminal damage-arson	8,420
Stalking involving serious alarm	1		0
Harassment–In fear of violence	3		0
Theft from dwelling	2	Domestic burglary	5,930
Coercive behavior	1		0
Criminal damage over 5000	1		0

In the control group, we have 268 offences in the 6 months and 367 offences in the 12 months. According to Table 10, CARA on average reduces the offences by 39% in the 6 months and 41% in the 12 months. The economic benefit calculations appear in Table 14 below.

**TABLE 14:** Economic benefits of the CARA service (Hampshire Constabulary).

		Number of crimes	Total cost of crime
Control Group	6 months	268	£1,528,375.49
	12 months	367	£2,092,961.96
Estimated costs after CARA	6 months	163.48	£932,309.05
	12 months	216.53	£1,234,847.56
CARA Benefit	6 months	104.52	£596,066.44
	12 months	150.47	£858,114.40

**TABLE 15:** Analysis of risk.

Risk level	(a) Five-level risk and needs system level II	(b) CARA standard ( = low) risk	(c) Five-level risk and needs system level III
Criminogenic needs	• One or two identifiable criminogenic needs • Needs are transitory or acute	• One or two identifiable criminogenic needs • Needs are transitory or acute	• Multiple criminogenic needs driven by one or two discrete criminogenic needs that are considered primary drivers of their criminal behavior
Non-criminogenic needs	• Some non-criminogenic needs, but these, too, would not be severe • Some identifiable resources and strengths	• Some non-criminogenic needs, but these, could be severe and include past trauma. • Some identifiable resources and strengths	• Some non-criminogenic needs typical of the general correctional population (e.g., past trauma or mental health needs). • Some identifiable resources and strengths

Consider the third column in Table 14. First, it contains the number of crimes/offences in the control group. Below that, it contains the predicted offences that we would have if the CARA treatment was applied to this control group. Then, further below, it reports the amount of reduction, which is the number of crimes reduction caused by CARA. For example, at the six-month interval, CARA is predicted to reduce the 268 crimes in the control group to 163.48 crimes, a reduction of 104.52 crimes. Therefore, the economic benefit of CARA is that it prevents 104.52 crimes, each of which costs £5,702.89. Therefore, the economic benefit of CARA is 104.52 × £5,702.89 = £596,066.

We now complete the calculations by taking into consideration the cost of the CARA workshops. In the control group, there are 309 individuals and the cost to put them through CARA would be £309 × £250 = £77,250. Therefore, the net benefit of CARA would be £596,066.44−£77,250 = £518,816.44 in a period of 6 months and £780,864.40 annually. The benefit-cost ratio is equal to 11.10, meaning that for each pound invested in CARA, there is an economic benefit of 11.10 pounds, annually.

## 5. Conclusion

We conducted a robust impact and economic evaluation. It showed that not only did CARA work in terms of reducing re-offending, but its economic benefit is also considerable saving £2.75 in West Midlands for every pound spent and £11.1 in Hampshire for every pound spent there. While the benefits vary, it is positive and economically significant across both areas.

This difference in magnitude could occur for several reasons; for example, variations in the socio-economic and demographic characteristics of the force areas. West Midlands has a young and diverse population but is also characterized by high levels of unemployment and significant economic and social deprivation and related inequalities.^9^ Hampshire on the other hand is one of the most affluent counties in England and nearly 90% of its population is White British.^10^ Future work may consider if CARA needs more tailoring to take account of these socio-economic and demographic factors to further improve effectiveness.

Thus, there appears to be a compelling case for CARA to be adopted nationally while also considering whether more tailoring and complementing with other types of support may make it have an even bigger impact.

## Data availability statement

The data analyzed in this study is subject to the following licenses/restrictions: The datasets analyzed for this study are proprietary to The West Midlands Police, the Hampshire Constabulary and The Hampton Trust, and have been shared with the University of Birmingham through information sharing agreements. Requests to access these datasets should be directed to The West Midlands Police, the Hampshire Constabulary, and The Hampton Trust.

## Ethics statement

The studies involving human participants were reviewed and approved by Humanities and Social Sciences Ethics Commitee, University of Birmingham. Written informed consent for participation was not required for this study in accordance with the National legislation and the institutional requirements.

## Author contributions

YK, SB, CC, CB-J, JT, EK, and HF have jointly participated in the following: study conception and design, data collection, analysis and interpretation of results, and manuscript preparation. All authors contributed to the article and approved the submitted version.

## Footnotes

1 The term “offender” for individuals selected for the CARA intervention means that these individuals have admitted the offence, but they have not necessarily been convicted in a court.

2 This is police terminology for low-risk cases. See Table 15 in the Supplementary materials for where the risk sits in with Hanson et al.’s (2017) risk levels.

3 In the following the terms “treatment” and “treatment group” are taken as synonymous to “intervention” and “intervention group.” We maintain the term “treatment” throughout because of its prevalence in statistical analysis which can refer to any intervention, be it a drug or an incentive payment or as on this case a BIP intervention. Indeed the “average treatment effect” (see Holland, 1986) is the technical term used to denote the difference in average outcomes in units assigned to the intervention vs. the control.

4 Statistical tests for differences in means or proportions can be found in Tables 2, 3, 5, 6.

5 We have repeated the analysis with these four cases excluded from the sample at the suggestion of a referee. The results are numerically almost identical.

6 There were 18 high-risk cases in the control and 4 high risk cases in the CARA treatment group. These cases were considered standard or medium risk at referral and/or at the time of the CARA workshops but were later upgraded to high. The results do not change if we exclude this group from the analysis given the very small number of such cases.

7 According to the Hampshire Constabulary, an individual can be shown as an offender without ever being arrested. This is because of a path which allows perpetrators to voluntarily attend CARA and thus avoid the process of an arrest.

8 Indeed, DVA is associated with increased incidence of not only mental ill health but also cardiovascular disease, diabetes, all-cause mortality and several chronic health conditions (Chandan et al., 2019a,b, 2020).

9 See https://www.wmca.org.uk/media/4290/state-of-the-region-2020-final-full-report.pdf

10 See https://documents.hants.gov.uk/Economy/ZCardHantswebFinal.pdf

## References

[B1] Andrews D. A. Bonta J. (2006). *The Psychology of Criminal Conduct*, 4th Edn. Newark, NJ: LexisNexis.

[B2] Andrews D. A. Bonta J. (2007). *Risk-Need-Responsivity Model for Offender Assessment and Rehabilitation. Her Majesty the Queen in Right of Canada.* Available online at: https://www.publicsafety.gc.ca/cnt/rsrcs/pblctns/rsk-nd-rspnsvty/index-en.aspx (accessed November 29, 2022).

[B3] Bonta J. Wallace-Capretta S. Rooney R. (2000). A quasi-experimental evaluation of an intensive rehabilitation supervision program. *Crim. Just. Behav.* 27 312–329. 10.1177/0093854800027003003

[B4] Chandan J. Thomas T. Bradbury-Jones C. Taylor J. Bandyopadhyay S. Nirantharakumar K. (2020). The risk of cardiometabolic disease and all-cause mortality in female survivors of domestic abuse. *J. Am. Heart Assoc.* 9:e014580. 10.1161/JAHA.119.014580 32063124 PMC7070197

[B5] Chandan J. Thomas T. Raza K. Bradbury-Jones C. Taylor J. Bandyopadhyay S. (2019a). Intimate partner violence and the risk of developing fibromyalgia and chronic fatigue syndrome. *J. Interpers. Violence* 36 N12279–N12298. 10.1177/0886260519888515 31805821

[B6] Chandan J. Thomas T. Bradbury-Jones C. Russell R. Bandyopadhyay S. Nirantharakumar K. (2019b). Female survivors of intimate partner violence and risk of depression, anxiety and serious mental illness: a retrospective cohort study using UK primary care records. *Br. J. Psychiatry* 217 1–6. 10.1192/bjp.2019.124 31171045

[B7] Christie C. Karavias Y. Bandyopadhyay S. Bradbury-Jones C. Taylor J. Kane E. (2022). The CARA (cautioning and relationship abuse) service theory of change, impact evaluation and economic benefits study report. *PsyArXiv* [Preprint]. 10.31234/osf.io/jw9uy

[B8] College of Policing (2020). *Authorised Professional Practice on Domestic Abuse.* Available online at: https://www.app.college.police.uk/app-content/major-investigation-and-public-protection/domestic-abuse/ (accessed June 10, 2022).

[B9] Cornelius N. (2013). *Perceptions of Domestic Abuse Victims to Police Disposals Post-Arrest by Conditional Caution, Simple Caution or No Further Action.* Unpublished thesis. Cambridge, MA: University of Cambridge.

[B10] Cox S. M. Rivolta P. M. (2021). Evaluative outcomes of connecticut’s batterer intervention for high risk offenders. *J. Aggress. Maltreat. Trauma* 30 931–949. 10.1080/10926771.2019.1581862

[B11] DASH (2009). Available online at: https://www.dashriskchecklist.co.uk/wp-content/uploads/2021/12/DASH-2009.pdf (accessed June 10, 2022).

[B12] Feder L. Wilson D. B. Austin S. (2008). Court-mandated interventions for individuals convicted of domestic violence. *Campbell Syst. Rev.* 2008 1–46. 10.4073/csr.2008.12 PMC8356297 37133255

[B13] Gibbs P. (2018). *Love, Fear and Control – Does the Criminal Justice System Reduce Domestic Abuse? Transform Justice, London.* Available online at: www.transformjustice.org.uk/wp-content/uploads/2018/09/TJ_August_WEB_V1.pdf (accessed November 29, 2022).

[B14] Gondolf E. W. (2012). *The Future of Batterer Programs: Reassessing Evidence-Based Practices.* Boston, MA: Northeastern University Press.

[B15] Hanson R. K. Bourgon G. McGrath R. J. Kroner D. D’Amora D. A. Thomas S. S. (2017). *A Five-Level Risk and Needs System: Maximizing Assessment Results in Corrections through the Development of a Common Language.* New York, NY: The Council of State Governments Justice Center, National Reentry Resource Center, and Public Safety Canada.

[B16] Heckman J. J. Ichimura H. Todd P. E. (1997). Matching as an econometric evaluation estimator: evidence from evaluationg a job training programme. *Rev. Econ. Stud.* 64 605–654. 10.2307/2971733

[B17] Heeks M. Reed S. Tafsiri M. Prince S. (2018). *The Economic and Social Costs of Crime.* London: Home Office.

[B18] Holland P. W. (1986). Statistics and causal inference. *J. Am. Stat. Assoc.* 81 945–960. 10.1080/01621459.1986.10478354

[B19] Jarman R. (2011). *Could Conditional Cautions be Used as a Suitable Intervention for Certain Cases of Domestic Violence? A Feasibility Study for Conducting a Randomised Controlled Trial in Hampshire.* Unpublished thesis. Cambridge, MA: University of Cambridge.

[B20] Maiuro R. D. Eberle J. A. (2008). State standards for domestic violence perpetrator treatment: current status, trends, and recommendations. *Violence Victims* 23 133–155. 10.1891/0886-6708.23.2.133 18624086

[B21] Office for National Statistics (2022a). *The Crime Survey for England and Wales: Year Ending March 2022.* Available online at: https://www.ons.gov.uk/peoplepopulationandcommunity/crimeandjustice/bulletins/crimeinenglandandwales/yearendingmarch2022#:~:text=Trends%20in%20police%20recorded%20crime&text=Police%20recorded%20crime%20in%20England,2020%20(6.1%20million%20offences) (accessed June 10, 2022).

[B22] Office for National Statistics (2022b). *Crime Severity Score index (CSS).* Available online at: https://www.ons.gov.uk/peoplepopulationandcommunity/crimeandjustice/datasets/crimeseverityscoreexperimentalstatistics (accessed June 10, 2022).

[B23] Rosenbaum P. R. (2002). Covariance adjustment in randomized experiments and observational studies. *Stat. Sci.* 17 286–327. 10.1214/ss/1042727942

[B24] Rosenbaum P. R. Rubin D. B. (1983). The central role of propensity score in observational studies for causal effects. *Biometrika* 70 41–55. 10.1093/biomet/70.1.41

[B25] Rowland J. (2013). *What Happens After Arrest for Domestic Abuse: A Prospective Longitudinal Analysis of Over 2,200 Cases.* Unpublished thesis. Cambridge, MA: University of Cambridge.

[B26] Sherman L. W. (2020). How to count crime: the cambridge harm index consensus. *Camb. J. Evid. Based Polic.* 4 1–14. 10.1007/s41887-020-00043-2

[B27] Strang H. Sherman L. Ariel B. Chilton S. Braddock R. Rowlinson T. (2017). Reducing the harm of intimate partner violence: randomized controlled trial of the hampshire constabulary CARA experiment. *Camb. J. Evid. Based Polic.* 1 160–173. 10.1007/s41887-017-0007-x

